# A dataset of human fMRI/MEG experiments with eye tracking for spatial memory research using virtual reality

**DOI:** 10.1016/j.dib.2022.108380

**Published:** 2022-06-14

**Authors:** Bo Zhang, Yuji Naya

**Affiliations:** a School of Psychological and Cognitive Sciences, Peking University, No. 52, Haidian Road, Haidian District, Beijing 100805, China; b IDG/McGovern Institute for Brain Research at Peking University, No. 52, Haidian Road, Haidian District, Beijing 100805, China; c Beijing Academy of Artificial Intelligence, Beijing 100084, China; d Tsinghua Laboratory of Brain and Intelligence, Tsinghua University, 160 Chengfu Rd., SanCaiTang Building, Haidian District, Beijing 100084, China; e Center for Life Sciences, Peking University, No. 52, Haidian Road, Haidian District, Beijing 100805, China; f Beijing Key Laboratory of Behavior and Mental Health, Peking University, No. 52, Haidian Road, Haidian District, Beijing 100805, China

**Keywords:** fMRI, MEG, Cognitive map, Navigation, Episodic memory, Parietal lobe, Medial temporal lobe, Egocentric space

## Abstract

A dataset consisting of whole-brain fMRI (functional magnetic resonance imaging)/MEG (magnetoencephalography) images, eye tracking files, and behavioral records from healthy adult human participants when they performed a spatial-memory paradigm in a virtual environment was collected to investigate the neural representation of the cognitive map defined by unique spatial relationship of three objects, as well as the neural dynamics of the cognitive map following the task demand from localizing self-location to remembering the target location relative to the self-body. The dataset, including both fMRI and MEG, was also used to investigate the neural networks involved in representing a target within and outside the visual field. The dataset included 19 and 12 university students at Peking University for fMRI and MEG experiments, respectively (fMRI: 12 women, 7 men; MEG: 4 women, 8 men). The average ages of those participants were 24.9 years (MRI: 18–30 years) and 22.5 years (MEG: 19–25 years), respectively. fMRI BOLD and T1-weighted images were acquired using a 3T Siemens Prisma scanner (Siemens, Erlangen, Germany) equipped with a 20-channel receiver head coil. MEG neuromagnetic data were acquired using a 275-channel MEG system (CTF MEG, Canada). The dataset could be further used to investigate a range of neural mechanisms involved in human spatial cognition or to develop a bioinspired deep neural network to enhance machines' abilities in spatial processing.

## Specifications Table

**Table:** 

Subject	Biological sciences, Neuroscience: Cognitive
Specific subject area	Neuroscience: Behavioral Neuroscience: Behavioral Neuroimaging, Cognitive Neuroscience
Type of data	Image
How the data were acquired	MRI T1-weighted and BOLD images were collected using a 3T Siemens Prisma scanner (Siemens, Erlangen, Germany) equipped with a 20-channel receiver head coil. MEG neuromagnetic data were collected with a 275-channel whole-head axial gradiometer DSQ-3500 MEG system (CTF MEG, Canada).
Data format	Raw
Description of data collection	MRI BOLD images were acquired with a multiband echo planar imaging sequence with a TR at 2 s and a multiband factor of 2, and T1-weighted anatomical data were acquired using MPRAGE sequence. MEG signals were acquired at a sampling rate of 1200 Hz.
Data source location	• Institution: Beijing Academy of Artificial Intelligence, Tsinghua University, Peking University • City/Town/Region: Haidian District, Beijing • Country: China
Data accessibility	Repository name: SCIENCE DATA BANK Data identification number: 10.11922/sciencedb.01460 Direct URL to data: https://www.scidb.cn/en/detail?dataSetId=c9a9f4695e1840499d904f8706ad093c
Related research article	**Zhang, B**., Wang, F., Zhang, Q., & Naya, Y. (2022). Distinct networks coupled with parietal cortex for spatial representations inside and outside the visual field. NeuroImage, 119041.

## Value of the Data

• The data can be used to investigate spatial memory-related neural principles from dynamic statistical parametric maps of global neural signals recorded by MRI with a high spatial resolution of 2 mm and MEG with a high temporal resolution of 1200 samples/s.
• The data can be used to investigate the behavioral principle of eye movement as well as the relationship between eye movements and neural signals in spatial cognition tasks.
• The data can be used for further analysis in addition to our recent publications [1,2]. Further investigation may focus on how the function of the frontal lobe differs from that of the medial temporal lobe in coding cognitive maps.
• The data can be used to assist in training bioinspired deep neural networks to enhance the spatial cognition of machines.

## 1 Data Description

**Fig. 1:**
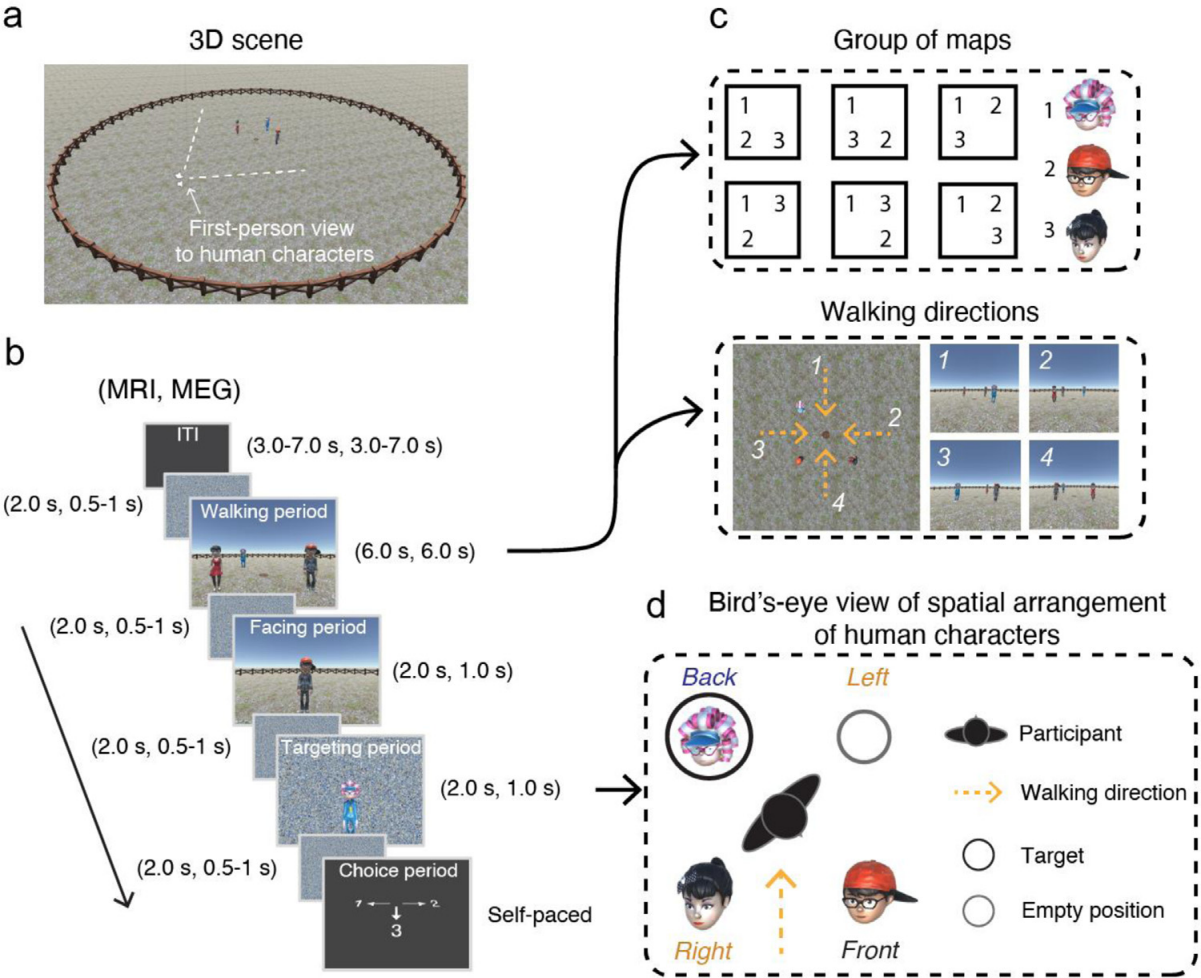
Illustration of the experimental design modified from [1,2]. (a) 3D environment of the SM task programmed using Unity Engine; (b) Experimental paradigm and timing for the MRI and MEG experiments. ITI (intertrial interval), walking period, facing period, targeting period, and choice period were included in each trial; (c) The first-person perspective visual stimuli in the walking period were determined by the maps (relative relationship of human characters) and the walking directions (orange arrow); (d) Egocentric-target conditions, the target would be located on the left, right, and back of the self-body of the participant. Note that participants never saw the environment from a bird's-eye view.

The data collected were the raw behavioral and neuroimaging files collected from our previous fMRI [1] and MEG [2] experiments. The two experiments were conducted independently based on a spatial-memory (SM) task with the same design except for the timing parameters (Fig. 1). In each experiment, three types of files were included: (1) text files, which stored the behavioral records (e.g., experimental conditions, types of stimuli, and participants’ responses) and neuroimaging timing files (i.e., the timing onsets of experimental stimuli); (2) neuroimaging files, which were either DICOM (Digital Imaging and Communications in Medicine) files generated by a MAGNETOM Prisma MRI scanner (Siemens Healthcare, Erlangen, Germany) with the file extension “.dcm” [3], or were generated by the MEG system (CTF Systems, Inc., Port Coquitlam, British Columbia, Canada) with the manufacturer specific file extension “.ds” [4,5]; and (3) eye-tracking files, which were acquired by an EyeLink 1000 plus (SR Research, Ottawa, ON, Canada) with the file extension “.edf” [8]. The files type could be identified by the keyword “fMRI” or “MEG” in the folder name. For example, the text files of the fMRI and MEG experiments can be found in the folder “fMRI_behavior” and “MEG_behavior”, respectively, while the folders “MRI_eyedata” and “MEG_eyedata” include eye-tracking files for the two experiments. Specificly, neuroimaging files were saved for each participant and each experimental type. For example, folder “MRI_Scanning_sub8” represents the fMRI data of Participant 8.

### 1.1 Text File

**Fig. 2:**
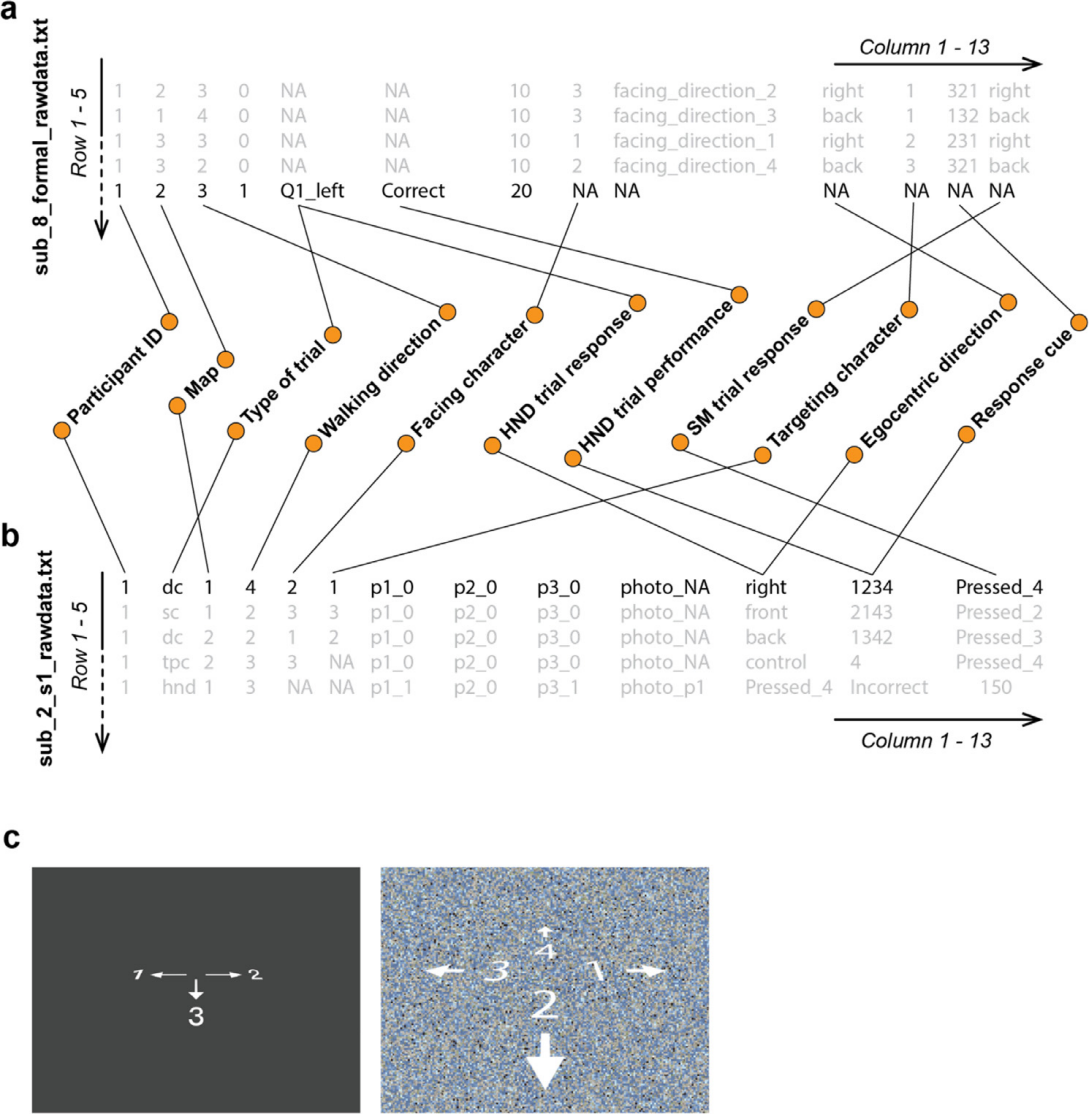
Examples of text files. The top 5 rows of “sub_8_formal_rawdata.txt” and “sub_2_s1_rawdata.txt” are listed as examples in (a) and (b), respectively. The label of each column is shown in the center. The rows highlighted by black color are the examples for an HND trial (a) and an SM trial (b), where “Q1_left” indicates that the participant pressed the “left” key; the designations dc/sc/fpc(not presented)/tpc are the abbreviation of “different character”/“same character”/“facing period control”/“targeting period control”, respectively, which indicate that the character presented during the target period is different/same as the character presented during the facing period, or no character was presented during the facing (fpc) or the targeting period (tpc). NA denotes “not available”; for example, there was no demand for participants to press the “left” or “right” key in the SM trial, and the records of Column 4 in (a) are labeled as NA for SM trials (Row 1–4). (c) shows the examples of response cues “231” (shown on left) and “4123” (shown on right) in the fMRI and MEG experiments, respectively. The records not clarified in the figure include the following: (a) Column 4 indicates that whether any character nodded their head, Column 7 indicates accumulated reward, and Column 9 indicates the participant's allocentric direction during the facing & targeting period; (b) Column 7–9 indicate which characters nodded their heads during the walking period, for example, character 1 & 3 nodded their heads in trial 1, and Column 8 indicates that the photo of character 1 was presented during the response period.

**Fig. 3:**
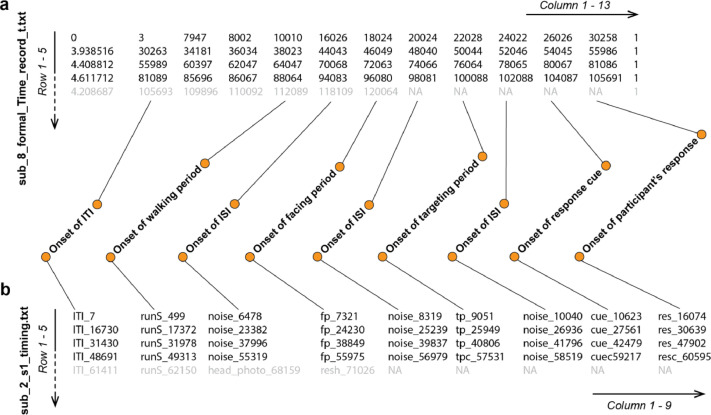
Example of timing files. The top 5 rows of “sub_8_formal_Time_record_t.txt” and “sub_2_s1_timing.txt” are listed as examples in (a) and (b), respectively. Numbers recorded by timing files represent the time onset of experimental periods in milliseconds. Labels of each experimental period of SM trials (highlighted by black color) are shown in the center. NA denotes “not available”, for example, there was no targeting period presented to participants in HND trials (Row 1-4). The timing records not clarified in (a) include the following: Column 1 indicates the duration of a 1.0-to-5.0 s random ITI, Columns 3 & 4 represent the onsets of a fixed 2.0 s period (a black screen) and a 2.0 s noise period (ISI, interstimuli interval), respectively, and Column 13 represents the # of experimental sessions. Please note that the first trial has a fixed 8.0 s ITI before the ISI.

Two types of text files (behavioral and timing files) were included for each participant and experiment, and the corresponding file names were defined by the Participant ID number and the type of text file. For example, behavioral files were labeled as “sub_#_formal_rawdata.txt”, while timing files were labeled as “sub_#_formal_Time_record_t.txt” (# denotes Participant ID number). For MEG, the text files were additionally labeled according to the # of experimental session. For example, files “sub_#_s1_rawdata.txt” and “sub_#_s1_timing.txt” indicate “session 1” for the given participant. In each text file, each row represents a trial, and each column represents the information of the experimental stimuli, the participant's response,or timing information for a given trial. Examples are given in Figs. 2 & 3.

### 1.2 Neuroimaging Files

All neuroimaging files include two types of files: one is a high-resolution T1-weighted image, which was stored in subfolder “T1” (i.e., “./MRI_Scanning_sub#/T1/”, # denotes Participant ID number), and the other type is a T2-weighted BOLD image, which was stored in subfolder “bold_run#” (# denotes experimental session). Both types of files are DICOM files (see [3] for details). Each file in subfolder “T1” includes one slice of high-resolution 3D brain, while each file in subfolder “bold_run#” includes the whole-brain bold signals recorded from a specific 2.0 s duration of an experiment (e.g., the file “NAYA10_MengTao-1006-sms_bold_run1-00003” represents Participant 10’s whole-brain bold signal recorded from the third 2.0 s duration from the onset of session 1). For MEG, neuromagnetic signals were stored in files with manufacturer specific extension “.ds” (see [4,5] for details), whose file names were defined according to the Participant ID number, project ID number, scanning date, and experimental session (e.g., the neuromagnetic file “S10_G14PKU_20190725_01.ds” indicates participant “10”, project “G14PKU”, scanning date “2019/07/25”, and session “1”). Similar to the fMRI data, each participant's folder also includes a T1-weighted image in folder “T1”.

### 1.3 Eye-Tracking Files

The file names of eye data are labeled by the keyword “el” (abbreviation of “EyeLink”) and the Participant ID number. For example, file “el_8.edf” indicates Participant 8. For MEG, eye data files are additionally labeled by the # of experimental session. For example, file “el_8_s1.edf” indicates Participant 8 and session 1. Eye-tracking files store messages (Event ID), participants’ button presses, and eye movement samples of the experiment; for details of eye-tracking files and their file structure, please refer to [8]). Due to technical issues, parts of the eye movements from five participants in the MEG experiment were not included in the present dataset, which included sessions 2–4 for Participant 2, sessions 3 and 4 for Participants 3 and 4, sessions 2 and 3 for Participant 6, and session 1 for Participant 13.

## 2 Experimental Design, Materials and Methods

### 2.1 Spatial-Memory (SM) Task

The data were collected when human participants performed an SM task [1,2], the task itself is a first-person perspective 3D game programmed by Unity Engine (Unity Technologies, San Francisco). In the game, three objects (human characters [Mixamo, San Francisco, https://www.mixamo.com]) were placed in the center of the circular environment (Fig. 1a), the spatial relationships of three characters formed six unique “maps” (Fig. 1c), and three of them were pseudorandomly selected for each participant as a group of “maps” in the experiment. In each trial, three sequential periods were included (Fig. 1b). Participants first encoded a map while walking from the environmental boundary toward the human characters (walking period), then participants were instructed that they were to stop at the center of the characters and used the cue character to localize their position relative to environment (facing period), and then localized a target character corresponding to their self-body by making self-paced responses (front, left, right, or back during the targeting period). Participants never saw a bird's-eye view of the virtual environment. In other words, they were blinded to the map concept throughout the task.

The main purpose of the SM task was the representational localization of a cognitive map, which is defined by the spatial relationship of characters in a 3D scene. To reduce the possibility that participants voluntarily memorized the spatial relationship of the object from a first-person perspective view, each of three characters was designed using animation to nod their head with 20.6% probability at a random time point during the walking period, and participants were required to pay attention to the heads of the human characters rather than to memorize their spatial arrangement during walking. The performance of participants was examined by dead-nodding detection (HND) trials (10% of experimental trials). In each trial, participants were required to indicate whether a given character presented on screen nodded its head or not. Note that the HND trials were indistinguishable from SM trials during the walking period, and both types of trials required participants to detect the head nodding of characters.

For the MEG experiment, two control conditions were additionally included by the spatial memory task. In the control condition, a white cross was presented instead of a human character either during both the facing and targeting periods or during the targeting period alone. In the response period, participants made a self-paced response to a random number presented on the screen. Both the fMRI and MEG experiments included four scanning sessions and took approximately 70 min. During scanning, experimental stimuli were rendered on a PC and presented on an LCD monitor with a screen resolution of 1024 × 768.

### 2.2 fMRI, MEG, Eye Data Acquisition and Parameters

All participants had corrected vision using MRI/MEG-compatible glasses or contact lenses and were required to stay still during the experiment. Their heads were stabilized either with a foam headrest in the MRI scanner or with chin and forehead rests in the MEG scanner. In the fMRI experiment, data were acquired by a 3T Siemens Prisma scanner (Siemens, Erlangen, Germany) equipped with a 20-channel receiver head coil. High-resolution T1-weighted images were acquired using a magnetization-prepared rapid gradient-echo (MP-RAGE) sequence with the following parameters: TR: 2530 ms; TE: 2.98 ms; matrix size: 448 × 512 × 192; flip angle: 7°; resolution: 0.5 × 0.5 × 1 mm^3^; number of slices: 192; slice thickness: 1 mm; slice orientation: sagittal. T2-weighted BOLD images were acquired with a multiband echo planar imaging (EPI) sequence with the following parameters: multiband factor: 2; TR: 2000 ms; TE: 30 ms; matrix size: 112 × 112 × 62; flip angle: 90°; gap: 0.3 mm; resolution: 2 × 2 × 2.3 mm^3^; number of slices: 62; slice thickness: 2 mm; gap between slices: 0.3 mm; slice orientation: transversal. In the MEG experiment, data were acquired by a 275-channel whole-head axial gradiometer DSQ-3500 MEG system (CTF MEG, Canada) [6], and a 1200 Hz sampling rate was used (see [2] for details of the experimental setup). High-resolution T1-weighted images for each participant in the MEG experiment were acquired from a 3T Siemens Prisma scanner with the following parameters: voxel size: 1 × 1 × 1 mm^3^; flip angle: 9°; TE: 1.97 ms; TR: 2,300 ms; field of view: 256 × 256 × 176 mm^3^. For both the fMRI and MEG experiments, eye data were recorded by an Eyelink 1000 Plus eye tracking system at 1000 Hz (SR Research Ltd., Mississauga, Canada). Eye movements were monitored monocularly from the dominant eye. Prior to each experimental session, a nine-point calibration and validation routine was performed if the error exceeded 1° of visual angle during a drift check. During eye tracking, a threshold of 30°/s for velocity and 8000°/s for acceleration of eye movement were used [7].

## Ethics Statements

This work recruited 19 and 12 university students from Peking University in the fMRI (12 females and 7 males aged from 18 to 30 years) and MEG experiments (4 females and 8 males aged from 19 to 25 years), respectively. None of the participants had a history of psychiatric or neurological disorders, and all participants were right-handed with normal or corrected-to-normal vision during the experiments. This work was approved by the Research Ethics Committee of Peking University, and was in accordance with the The Code of Ethics of the World Medical Association (Declaration of Helsinki), the corresponding protocol number is #2017-07-06.

## CRediT authorship contribution statement

**Bo Zhang:** Conceptualization, Methodology, Data curation, Software, Writing – original draft, Visualization. **Yuji Naya:** Conceptualization, Resources, Validation, Writing – review & editing, Supervision, Project administration, Funding acquisition.

## Declaration of Competing Interest

The authors declare that they have no known competing financial interests or personal relationships that could have appeared to influence the work reported in this paper.

## Data Availability

A dataset of human fMRI/MEG experiments with eye tracking for spatial memory research using virtual reality (Original data) (SCIENCE DATA BANK).
